# Wie gut sind Patienten mit entzündlich rheumatischen Erkrankungen gegen Masern geschützt?

**DOI:** 10.1007/s00393-020-00874-4

**Published:** 2020-09-15

**Authors:** U. Kiltz, A. Celik, S. Tsiami, X. Baraliakos, I. Andreica, D. Kiefer, B. Bühring, J. Braun

**Affiliations:** 1 Rheumazentrum Ruhrgebiet, Herne und Ruhr-Universität Bochum, St. Elisabeth Gruppe GmbH, Claudiusstr. 45, 44649 Herne, Deutschland; 2 Klinikum Westfalen, Dortmund, Deutschland

**Keywords:** Infektionskrankheiten, Masern, Impfung, Protektive Antikörper, Masernschutzgesetz, Infectious diseases, Measles, Vaccination, Protective antibodies, Measles Protection Act

## Abstract

**Hintergrund:**

Patienten mit entzündlich rheumatischen Erkrankungen haben aufgrund ihrer Autoimmunerkrankung, aber auch bedingt durch die immunsuppressive Medikation ein erhöhtes Infektrisiko. Obwohl Impfungen in der Primärprophylaxe von Infektionen bekanntermaßen effektiv sind, ist die Impfrate in Deutschland generell zu niedrig. Wegen des zuletzt zunehmenden, teils epidemieartigen Auftretens von Masern ist die Lebendimpfung gegen Masern in Deutschland seit Kurzem gesetzlich vorgeschrieben.

**Fragestellung:**

Wie viele Patienten mit entzündlich rheumatischen Erkrankungen sind aktuell ausreichend gegen Masern geschützt?

**Methode:**

Patienten mit entzündlich rheumatischen Erkrankungen des Rheumazentrums Ruhrgebiet wurden zwischen Dezember 2017 und Oktober 2018 prospektiv und konsekutiv eingeschlossen. Dabei wurden Daten zu Erkrankung und Therapie auf Ebene von Substanzklassen sowie die Impf- und Infektanamnese erhoben. Alle Angaben zu Impfungen wurden im Impfpass kontrolliert. Antikörpertiter gegen Masern wurden mit ELISA bestimmt. Als Schwellenwert für einen ausreichenden Schutz gegen Masern wurden 150 mIU/ml festgelegt.

**Ergebnis:**

Von 975 Patienten konnten 540 (55,4 %) einen Impfausweis vorlegen. Bei 201 Patienten mit Ausweis (37,2 %) lagen dokumentierte Impfungen seit Geburt vor. Insgesamt hatten 45 von 267 nach 1970 geborene Patienten (16,9 %) einen suffizienten Impfschutz gegen Masern. Die anamnestischen Angaben zu einer Masernerkrankung in der Kindheit differenzierten nicht zwischen Patienten mit und ohne protektiven Masern-IgG-Antikörpern. Protektive Masern-IgG-Antikörper wurden bei 901 Patienten von 928 Patienten mit Messung der Masern-IgG-Antikörperspiegel (97,1 %) nachgewiesen. Die unterschiedlichen Wirkprinzipien der aktuellen immunsuppressiven Therapie hatten darauf keinen Einfluss.

**Diskussion:**

Diese Daten zeigen, dass mindestens 2,9 % der Patienten keinen ausreichenden Schutz gegen Masern haben. Interessanterweise hatte die Mehrheit der nach 1970 geborenen Patienten protektive Antikörper trotz fehlenden Impfschutzes gegen Masern. Die Anstrengungen sowohl im primär- als auch im fachärztlichen Bereich sollten dringend verstärkt werden, um eine adäquate Infektionsprophylaxe bei besonders gefährdeten Patienten gewährleisten zu können.

## Abstract (en)

**Background:**

Patients with inflammatory rheumatic diseases have an increased risk of infections due to the autoimmune disease but also due to the immunosuppressive medication. Although vaccinations are known to be effective in the primary prophylaxis of infections, the vaccination rate in Germany is generally too low. Due to the recently increasing, sometimes epidemic-like occurrence of measles, the administration of live vaccine against measles has recently become required by law.

**Objective:**

How many patients with inflammatory rheumatic diseases are currently sufficiently protected against measles?

**Method:**

Between December 2017 and October 2018 patients with inflammatory rheumatic diseases at the Ruhrgebiet Rheumatism Center were prospectively and consecutively included. Data on the disease and treatment at the level of substance classes, patient history of vaccination and infections were collated. All information on vaccinations were controlled in the vaccination certificate. Antibodies against measles were determined using ELISA. The threshold for sufficient protection against measles was set at 150 mIU/ml.

**Results:**

Out of 975 patients 540 (55.4%) could present a vaccination certificate. In 201 patients with a certificate (37.2%) vaccination had been documented since birth. Overall, 45 out of 267 patients born after 1970 (16.9%) had sufficient protection against measles. The patient history of measles in childhood showed no differences between patients with and without protective measles IgG antibodies. Protective measles IgG antibodies were detected in 901 out of 928 patients with measurement of the measles IgG antibody level (97.1%). The different principles of action of the current immunosuppressive treatments had no influence on this.

**Conclusion:**

These data show that at least 2.9% of the patients did not have sufficient protection against measles. Interestingly, the majority of patients born after 1970 had protective antibodies despite the lack of vaccination against measles. The efforts in primary and also in the specialist medical care should be urgently strengthened in order to be able to guarantee an adequate infection prophylaxis in particularly endangered patients.

Die Masern sind eine seit dem 7. Jahrhundert bekannte Infektionskrankheit des Menschen, die durch das Masernvirus (MV) verursacht wird. Das MV, ein Paramyxovirus der Gattung Morbillivirus, hat einen Kern aus einsträngiger Ribonukleinsäure (RNA). Vor der Einführung eines wirksamen Masernimpfstoffs erkrankte praktisch jeder Mensch schon in der Kindheit an Masern. Die Diagnose einer Maserninfektion wird klinisch gestellt [1, 2]. Durch die aktuell verfügbaren Impfstoffe, die einen abgeschwächten MV-Lebendimpfstamm enthalten, wurden erhebliche Fortschritte im Hinblick auf die weltweite Impfdichte erzielt, was einen Rückgang der Inzidenz von Masern bewirkt hat [1, 2]. Allerdings hat sich die endemische Übertragung in vielen Teilen der Welt fortgesetzt. Die Schätzungen der Sterblichkeitsquote schwanken zwischen <0,01 % in Industrieländern und >5 % in Entwicklungsländern [1–3].

In Europa erkrankten im Jahr 2018 laut WHO 87.500 Menschen an Masern, 61 % der Betroffenen wurden hospitalisiert, in 72 Fällen verlief die Erkrankung tödlich (0,08 %). Die Gesamtzahl der 2018 mit dem MV infizierten Personen war die bislang höchste in diesem Jahrzehnt und ist im Jahr 2019 noch einmal angestiegen auf 90.000 Fälle in den ersten 6 Monaten [1, 3, 4]. Damit ist insgesamt nach wie vor von einer potenziell bedrohlichen Situation auszugehen, auch wenn die Zahl der Masernfälle in Deutschland im Jahr 2018 nach dem Bericht der Nationalen Verifizierungskommission Masern Röteln (NAVKO) im Vergleich zum Vorjahr etwas gesunken sind: in 2018 wurden 543 und in 2017 929 Masernfälle (Inzidenz: 5,8 bzw. 10,5/10^6^ Einwohner) gemeldet [5, 6]. Der von der WHO vorgegebene Indikator für eine erfolgreiche Masernelimination liegt bei einer Inzidenz von <1 Erkrankung pro 1 Mio. Einwohner und wird in Deutschland nicht erreicht [7, 8].

Aufgrund der Verfügbarkeit hochwirksamer und relativ kostengünstiger Impfstoffe, der Monotypizität des Virus und des Fehlens eines Tierreservoirs werden die Masern als geeigneter Kandidat für eine Eradikation und als essenziell für eine wirksame Infektionskontrollstrategie betrachtet [9]. Die Masernimpfung ist in Deutschland seit 1970 fester Bestandteil der Empfehlungen der Nationalen Kommission für Impfungen (STIKO) [10]. Nach den Vorgaben des Robert Koch-Instituts (RKI) soll die Kontrolle der Masernimmunität grundsätzlich durch Kontrolle des Impfausweises erfolgen [11]. Danach kann von einem zuverlässigen Schutz gegen Masern ausgegangen werden, wenn bei Kindern mindestens 2 oder bei Erwachsenen mindestens 1 MMR- oder Masernimpfungen im Impfpass dokumentiert sind. Eine Antikörperkontrolle auf Masern-IgG-Antikörper ist laut RKI normalerweise nicht erforderlich und wird auch nicht empfohlen [12]. Allerdings sagt das RKI auch klar, dass bei positivem Nachweis von Anti-Masern-IgG-Antikörpern grundsätzlich von Immunität d. h. von einem Schutz gegen Masern ausgegangen werden kann [8].

Obwohl in allen internationalen Leitlinien Impfungen gegen MV empfohlen werden, ist die Impfrate sowohl in Amerika als auch in Europa immer noch suboptimal [13–18]. Dies liegt zum Teil auch an nicht ausreichender Information der Bevölkerung, aber auch des medizinischen Personals und von Medizinstudenten [19, 20]. In Deutschland waren im Jahr 2010 insgesamt nur 91,5 % der einzuschulenden Kinder 2‑mal gegen Masern geimpft [20]. Im Vergleich der einzelnen Bundesländer lagen die Impfquoten zwischen 87,6 und 95,3 % [20]. Seit dem 01.03.2020 ist die Lebendimpfung gegen Masern in Deutschland gesetzlich vorgeschrieben [12]. Alle nach dem 31.12.1970 geborenen Personen, die in einer Gemeinschaftseinrichtung betreut werden, müssen einen Masernschutz nachweisen [21]. Personen, die in Gesundheitseinrichtungen wie Krankenhäusern und Arztpraxen oder in Gemeinschaftseinrichtungen oder Gemeinschaftsunterkünften tätig sind, sind ebenfalls verpflichtet einen Masernschutz nachzuweisen. Für Kinder, die bereits vor dem 01.03.2020 einen Kindergarten oder eine Schule besuchen, sowie für Beschäftigte in Gemeinschafts- und Gesundheitseinrichtungen gilt eine Nachweisfrist bis 31.07.2021.

Patienten mit entzündlich rheumatischen Erkrankungen wie der rheumatoiden Arthritis (RA) haben ein erhöhtes Risiko für infektiöse Morbidität und Mortalität aufgrund krankheitsbedingter Anomalien des Immunsystems und der Einnahme von immunsuppressiven Medikamenten wie der verschiedenen Klassen von „disease modifying anti-rheumatic drugs“ (DMARDs) [22]. Darüber hinaus sind mit der zunehmenden Zahl der bei RA-Patienten eingesetzten immunmodulierenden Medikamente die Sicherheit und Wirksamkeit von Impfungen bei solchen Therapien infrage gestellt [23]. Bei Immunsupprimierten oder bei Patienten mit zellulären Immundefekten verläuft die Maserninfektion ansonsten zwar nach außen hin schwach – das Masernexanthem tritt nicht oder nur atypisch in Erscheinung –, dagegen können sich als schwere Organkomplikationen eine progrediente Riesenzellpneumonie oder die Maserneinschlusskörperenzephalitis (MIBE) entwickeln, die mit einer Letalität von etwa 30 % einhergehen [1, 11].

Die STIKO hat Empfehlungen für Impfungen bei Patienten mit einer Immunschwäche veröffentlicht [24, 25]. Eine Impfung kann bei Patienten mit bestimmten Formen der Immundefizienz in Betracht gezogen werden, wenn der Nutzen der Impfung die Risiken überwiegt (ggf. Aufklärung und Dokumentation bei Off-label-Einsatz der Impfstoffe). Die MMR-Impfung ist für Personen mit „schwerer Immundefizienz“ generell kontraindiziert. Die altersentsprechende Grundimmunisierung sollte nach den Empfehlungen der STIKO daher vor Einleitung einer immunsuppressiven Therapie abgeschlossen sein bzw. möglichst bei nichtaktiver Grunderkrankung erfolgen. Wurde vor Beginn der Therapie in der Kindheit nur einmal gegen MMR geimpft, sollte die Komplettierung der Impfserie altersentsprechend abgeschlossen werden. Wenn eine Impfung nicht möglich ist und anhand des Impfpasses oder der ärztlichen Dokumentation nicht von einem Schutz ausgegangen werden kann, sollte eine serologische Kontrolle erfolgen, die Aufschluss darüber geben kann, ob bereits ein ausreichender Schutz besteht. Eine niedrig dosierte Glukokortikoidtherapie stellt für keinen der verfügbaren Impfstoffe eine Kontraindikation dar, für die anderen DMARDs und Immunsuppressiva ist auf die Empfehlungen der STIKO sowie international der EULAR zu verweisen [16, 25].

Interessanterweise zeigen einzelne, kleinere Studien zu Personen mit juveniler idiopathischer Arthritis (JIA) eine gute Sicherheit und Effektivität der MMR-Impfung [26, 27]. Nach Kenntnisstand der Autoren und mündlicher Rückfrage beim RKI gab es bisher bei Patienten mit rheumatischen Erkrankungen auch noch keinen Todesfall durch eine Masernimpfung. Gesunde Kontaktpersonen können dagegen ohne Gefahr für den rheumatologischen Patienten gegen Masern geimpft werden.

Die Deutsche Gesellschaft für Rheumatologie (DGRh) hat diese Empfehlungen für Patienten mit chronisch entzündlich rheumatischen Erkrankungen entsprechend umgesetzt [28, 29]. Der Impfstatus von Patienten mit rheumatischen Erkrankungen ist von zunehmender Bedeutung in der routinemäßigen Patientenversorgung, da einige der kürzlich zugelassenen Medikamente die Stärke der Immunantwort auf die Impfung beeinflussen können. Über den aktuellen Impfstatus und die Bereitschaft von Patienten mit rheumatischen Erkrankungen, sich in Deutschland impfen zu lassen, gibt es nur begrenzt Informationen. Auch fehlen epidemiologische Daten zur Umsetzung der Impfempfehlungen auf Ebene der Hausärzte. In einer prospektiven Studie haben wir vor einigen Jahren die Wirksamkeit standardisierter Impfempfehlungen für verschiedene Patientengruppen mit rheumatischen, in einem rheumatologischen Fachkrankenhaus behandelten Erkrankungen untersucht und berichtet, dass zu wenig empfohlene Impfungen in der täglichen Praxis auch durchgeführt werden [30]. Dies wurde in anderen Untersuchungen in anderen Regionen Deutschlands später bestätigt [31, 32].

Im Rahmen der Aktualisierung der 2011 zuerst veröffentlichten Empfehlungen der Europäischen Liga gegen Rheuma (EULAR) für die Impfung erwachsener Patienten mit autoimmun bedingten entzündlich rheumatischen Erkrankungen (AIIRD) wurden 4 systematische Literaturübersichten zu folgenden Themen durchgeführt: Inzidenz/Prävalenz von durch Impfung vermeidbaren Infektionen bei Patienten mit AIIRD; Wirksamkeit, Immunogenität und Sicherheit von Impfstoffen; Einfluss von DMARDs auf die Impfantwort; Wirkung der Impfung von Haushalten mit AIIRD-Patienten [15, 16]. Die aktualisierten EULAR-Empfehlungen zur Impfung erwachsener Patienten mit AIIRD umfassen 6 übergreifende Prinzipien und 9 Empfehlungen [16]. Die Ersteren beziehen sich auf die Notwendigkeit einer jährlichen Bewertung des Impfstatus, die gemeinsame Entscheidungsfindung und den Zeitpunkt der Impfung, wobei die Impfung während der Phase mit niedriger Krankheitsaktivität, vorzugsweise vor Beginn der Immunsuppression, bevorzugt wird.

In der hier vorliegenden prospektiven Studie der Routineversorgung eines rheumatologischen Fachkrankenhauses wurde untersucht, welcher Impfschutz gegen das MV bei Patienten mit entzündlich rheumatischen Erkrankungen vorliegt und welche Konsequenzen aufgrund der aktuellen Gesetzeslage daraus gezogen werden können.

## Methoden

Patienten mit entzündlich rheumatischen Erkrankungen des Rheumazentrum Ruhrgebiet wurden zwischen Dezember 2017 und Oktober 2018 prospektiv und konsekutiv eingeschlossen (Ethikvotum durch Ethikkommission der Ärztekammer Westfalen-Lippe, 2017-637-f-S). Die Untersuchung erfolgte einmalig am Tag einer ambulanten Routineuntersuchung, wobei Daten zu Erkrankung und Therapie auf Ebene von Substanzklassen sowie die Impf- und Infektanamnese erhoben wurden. Die Patienten wurden ebenfalls gefragt, ob sie von einem Arzt über die Bedeutung von Impfungen früher informiert worden sind (sog. Impfberatung). Es wurden bei allen Patienten folgende Parameter erhoben: demografische und klinische Angaben (Alter, Geschlecht, Diagnose, Krankheitsdauer, Komorbiditäten), Erfassung der Krankheitsaktivität (DAS-28, BASDAI und ASDAS je nach Art der Erkrankung) sowie der körperlichen Funktionsfähigkeit (Funktionsfragebogen Hannover [FFbH] für alle Erkrankungen bis auf Patienten mit axialer Spondyloarthritis, hier wurde der BASFI erhoben) sowie Art und Umfang der aktuellen Therapie. Die Umrechnung der FFbH-Werte erfolgte zur besseren Vergleichbarkeit in die Werte des international gebräuchlichen Health Assessment Questionnaire (HAQ) [33]. Alle Angaben zu Impfungen wurden im Impfpass kontrolliert.

Antikörpertiter gegen Masern wurden mit einem ELISA der Firma SIEMENS Enzygnost Anti-Measles IgG bestimmt. Als Schwellenwert für einen ausreichenden Schutz gegen Masern sind 150 mIU/ml für diesen ELISA durch den Hersteller festgelegt.

## Ergebnisse

Die klinischen Charakteristika der Patienten (*n* = 975) und ihre Impfergebnisse sind der Tab. 1 zu entnehmen. Etwa zwei Drittel unserer Patienten wurden vor 1970 geboren, sodass bei diesen Patienten von einer hohen Durchseuchung mit Masern in der Kindheit auszugehen ist.

**Table 1:** 

Variable	RA (*n* = 424)	axSpA (*n* = 145)	PsA (*n* = 132)	SLE (*n* = 41)	Andere Erkrankungen (*n* = 233)	Gesamte Kohorte (*n* = 975)
Alter, Jahre, Mittelwert (SD)	60,7 (13,4)	43,7 (12,4)	51,3 (12,7)	48,4 (17,6)	56,1 (16,9)	55,3 (15,5)
Geschlecht, männlich, *n* (%)	137 (32,3)	95 (65,5)	55 (41,7)	4 (9,8)	64 (27,5)	355 (36,4)
Aktueller Einsatz von bDMARDs, *n* (%)	163 (38,4)	103 (71,0)	76 (57,6)	9 (22,0)	59 (25,3)	410 (42,1)
Körperliche Funktion^a^	1,30 (0,76)^a^	4,0 (2,55)^b^	1,28 (0,68)	1,12 (0,67)	1,03 (0,69)	1,2 (0,7) (*n* = 830)^c^
CRP (mg/dl), Median (IQR)	0,3 (0,1–0,7)	0,2 (0,1–0,6)	0,2 (0,1–0,7)	0,2 (0,0–0,4)	0,3 (0,1–0,6)	0,2 (0,1–0,6)
Impfausweis vorhanden, *n* (%)	230 (54,2)	76 (52,4)	66 (50,0)	28 (68,3)	140 (60,1)	540 (55,4)
Patientenschulung bezüglich Impfungen, *n* (%)	273 (64,4)	101 (69,7)	81 (61,4)	28 (68,3)	146 (62,7)	629 (65,5)
Masernantikörper (mIU/ml), median (IQR)	8100 (4100–12.000)	4500 (1750–11.000)	7000 (3300–11.000)	7400 (2475–11.500)	7300 (3550–12.000)	7900 (2600–12.000)

*bDMARDs* biologische krankheitsmodifizierende („disease modifying“) antirheumatische Medikamente („antirheumatic drugs“), d. h. Biologika, *RA* rheumatoide Arthritis, *axSpA* axiale Spondyloarthritis, *PsA* Psoriasisarthritis, *SLE* systemischer Lupus erythematodes, *SD* xxx, *CRP* xxx, *IQR* xxx,

^a^Erhoben mit dem Funktionsfragebogen Hannover (FFbH) und Umrechnung in Werte des Health Assessment Questionnaire (HAQ)

^b^„Bath ankylosing spondylitis functional index“ (BASFI)

^c^Basierend auf der Gesamtkohorte ohne axSpA-Patienten

**Table 2:** 

	bDMARD-Gruppe (*n* = 499)	Kontrollgruppe (*n* = 476)
Alter, in Jahren, MW (SD)	52,9 (15,2)	57,7 (5,5)
Geschlecht, männlich, *n* (%)	196 (39,3 %)	159 (33,4 %)
Impfausweis vorliegend, *n* (%)	319 (63,9 %)	221 (46,4 %)
Anzahl Masernimpfungen bei 1970 geborenen Patienten (*n* = 133)	1,0 (0,9) (*n* = 77)	0,9 (0,9) (*n* = 56)
Vollständige Masernimpfung, *n* (%) (*n* = 45)	26 (33,8) (*n* = 26)	(33,9) (*n* = 19)
Masernantikörpertiter, mIU/ml, MW (SD)	8369 (5733)	8044 (5104)
Anzahl der Patienten ohne protektive Masernantikörper, *n* (%) (*n* = 928)	17 (3,5) (*n* = 481)	9 (2,0) (*n* = 447)
Impfberatung, *n* (%)	348 (69,7)	291 (61,1)

*bDMARDs* biologische krankheitsmodifizierende („disease modifying“) antirheumatische Medikamente („antirheumatic drugs“), d. h. Biologika, *MW* xxx, *SD* xxx

Insgesamt berichteten 446 Patienten (45,7 %), früher an Masern erkrankt gewesen zu sein, während 147 (15,1 %) angaben, keine Maserninfektion gehabt zu haben (zusammen *n* = 593), und 382 (39,2 %) konnten diesbezüglich gar keine Angaben machen. Das bedeutet, dass es bei 529 Patienten rein anamnestisch unklar war, ob ein Schutz gegen Masern aufgrund einer durchgemachten Infektion bestand.

## Datenlage nach Impfpass

**Figure 1:**
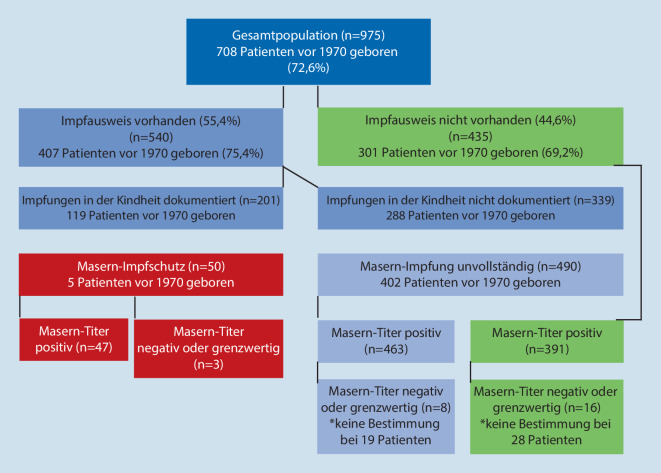

Von den 975 Patienten der Gesamtpopulation konnten 540 (55,4 %) einen Impfausweis vorlegen, aber nur bei 201 Patienten mit Impfausweis (37,2 %) lagen schriftlich dokumentierte Masernimpfungen seit Geburt vor (Abb. 1). Hiervon waren 94 Patienten mindestens 1‑mal gegen Masern geimpft worden (davon 80 Patienten, die nach 1970 geboren wurden). Von den 267 Patienten, die nach 1970 geboren wurden, lagen dokumentierte Masernimpfungen bei 133 Patienten (49,8 %) vor, bei 134 war dies nicht der Fall (50,2 %).

Bei den nach 1970 geborenen 143 Patienten mit Impfausweis waren bei 2 Patienten 3 (1,4 %), bei 43 Patienten 2 (30,1 %) und bei 35 Patienten 1 (24,5 %) Masernimpfung dokumentiert. Hieraus kann geschlossen werden, dass bei Patienten mit Impfpass ein Drittel (45 von 133 Patienten) einen ausreichenden Impfschutz gegen Masern aufwies (33,8 %), wohingegen in der Gesamtkohorte der nach 1970 geborenen Patienten nur bei 45 von 267 Patienten (16,9 %) ein ausreichender Impfschutz gegen Masern vorlag.

In der Kohorte waren aber auch insgesamt 14 Patienten, die im Erwachsenenalter gegen Masern immunisiert worden waren (1 Patient mit 4 Impfungen, 1 Patient mit 3 Impfungen, 3 Patienten mit 2 Impfungen und 9 Patienten mit 1 Impfung). Das bedeutet, dass nach „Aktenlage“ gemäß Impfpass nur insgesamt 59 von 267 Patienten (22,1 %) einen effektiven Impfschutz gegen Masern hatten.

## Datenlage nach protektiven Antikörpern

**Figure 2:**
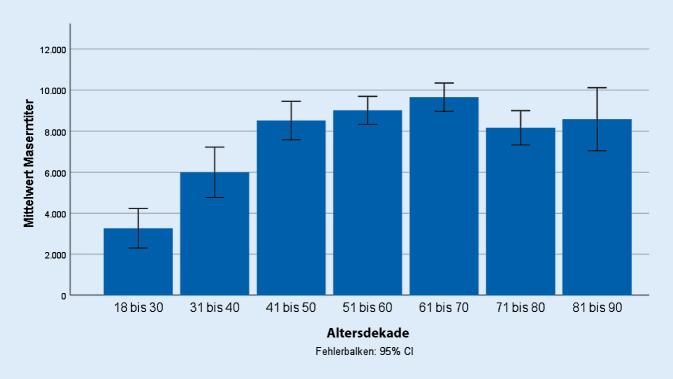

Es wurden IgG-Antikörper gegen Masern bei insgesamt 928 Patienten bestimmt (95,2 %). Protektive Masern-IgG-Antikörper wurden bei 901 Patienten (97,1 % bzw. 92,4 %) nachgewiesen (Titer 8183 ± 5420 mIU/ml [Spannbreite 330–26.000 mIU/ml], Abb. 2). Die Höhe der Titer sowie die Spannbreite der Titer unterschieden sich nicht zwischen den einzelnen Erkrankungen. Bei nach 1970 geborenen Patienten lag der Masernantikörpertiter (*n* = 235) mit im Mittel mit 5840 ± 5728 mIU/ml niedriger als bei den vor 1970 geborenen Patienten (*n* = 666) mit 9047 ± 5075 mIU/ml (*p* < 0,001).

Bei 15 bzw. 12 Patienten lag ein grenzwertiger bzw. negativer Titer vor (4,8 %). Der überwiegende Teil der Patienten (47/55), die nach Impfpass mindestens 2‑mal geimpft wurden, wiesen einen protektiven Maserntiter auf (85,4 %). Von den 930 Patienten ohne dokumentierte Masernimpfung bzw. ausreichende Masernimpfung wiesen 854 Patienten (91,8 %) einen protektiven Maserntiter auf.

Von den 901 Patienten mit nachgewiesenem Masern-IgG-Titer hatten 60 Patienten (6,7 %) einen relativ niedrigen Titer <1000 mIU/ml, Mittelwert 600 mIU/ml, bei Werten ≥1000 mIU/ml lag der Mittelwert bei 8000 mIU/ml.

Von den nach 1970 geborenen Patienten mit Impfausweis (*n* = 133) hatten 59 einen dokumentierten Impfschutz (44,3 %), davon hatten 3 keine protektiven Antikörper (5,1 %). Ein protektiver IgG-Antikörpertiter gegen Masern wurde jedoch bei 235 Patienten (88 %) festgestellt. Da zusätzlich 3 Patienten zwar keinen ausreichenden Maserntiter, aber eine 2‑fache Masernimpfung dokumentiert hatten, waren formal 238 Patienten geschützt (89,1 %).

Von 88 nach 1970 geborenen Patienten ohne zweifache Masernimpfung hatten 79 einen protektiven Antikörpertiter gegen Masern (89,8 %), bei 5 Patienten fehlte die Bestimmung. Daraus folgt, dass nur 4 nach 1970 geborene Patienten gänzlich ungeschützt waren, d. h. sie hatten weder einen protektiven Antikörpertiter noch eine ausreichende Anzahl an Masernimpfungen. Von den 32 Patienten ohne protektive IgG-Antikörpertiter gegen Masern hatten 12 einen grenzwertigen und 9 Patienten keinen Titer, und bei 11 war keine Bestimmung erfolgt.

## Datenlage nach durchgemachter Masernerkrankung

**Figure 3:**
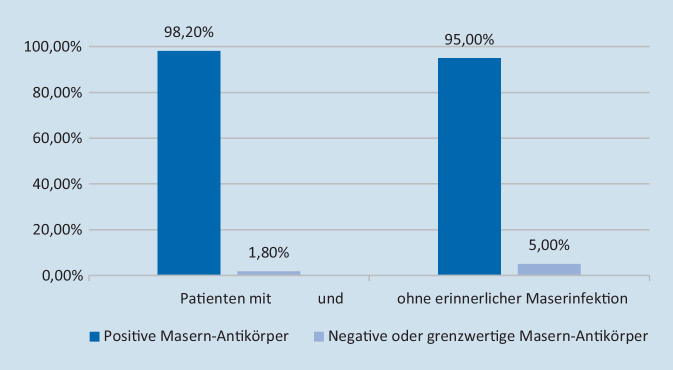

Von den 464 Patienten, die sich an eine Masernerkrankung erinnern konnten und bei denen Antikörper bestimmt worden waren (*n* = 440), hatten 8 keine nachweisbaren Masernantikörper (1,8 %) (Abb. 3). Die hohe Prävalenz an protektiven Antikörper lag allerdings auch bei Patienten vor, die angaben, dass sie keine Maserninfektion in der Kindheit durchgemacht hatten (Abb. 3). Allerdings lag bei allen Patienten, die angegeben hatten, weder an Masern erkrankt gewesen noch gegen Masern geimpft worden zu sein (*n* = 82), bei 71 (86,6 %) Patienten ein protektiver Masern-IgG-Antikörper vor, nur bei 4 Patienten fehlte die Antikörperbestimmung.

Bei den 12 Patienten mit negativem Masernantikörpertiter verneinten 3 Patienten eine Maserninfektion in der Kindheit, 6 konnten sich nicht erinnern, und 3 hatten in der Kindheit Masern gehabt.

Bei Patienten ohne erinnerliche Maserninfektion in der Kindheit (*n* = 147) lag der Masernantikörpertiter mit im Mittel 6757 ± 5519 mIU/ml niedriger als bei denen mit erinnerter Maserninfektion in der Kindheit (*n* = 464) mit 8934 ± 5320 mIU/ml (*p* < 0,001).

Interessanterweise war der Masernantikörpertiter bei Patienten mit ausreichendem Masernimpfschutz (*n* = 59) mit im Mittel mit 3361 ± 3418 mIU/ml deutlich niedriger als bei Patienten ohne ausreichenden Masernimpfschutz (*n* = 471) mit 8912 ± 5285 mIU/ml (*p* = <0,001). Allerdings ist die Wertigkeit der Impfanamnese zweifelhaft, da auch bei Pateinten, die eine Maserninfektion in der Kindheit explizit verneinten (*n* = 147), bei 95,0 % der Patienten ein protektiver Masernantikörper vorlag (Abb. 1).

Die aktuell vorliegende immunsuppressive Therapie hatte keinen Einfluss auf die Antikörpertiter. Bei Patienten unter bDMARD-Therapie zeigte sich zwar kein Unterschied hinsichtlich der Höhe der Masern-IgG-Antikörper, aber diese Patienten konnten häufiger einen Impfausweis vorlegen als Patienten ohne eine solche Therapie (63,9 vs. 46,4 %) (Tab. 2). Die Anzahl von mindestens 2 dokumentierten Masernimpfungen im Impfausweis unterschied sich zwischen Patienten mit und ohne bDMARD-Therapie nicht. Ungefähr gleich viele Patienten in beiden Gruppen konnten sich erinnern, durch Ärzte über Impfungen besonders informiert worden zu sein: unter bDMARDs 69,7 % vs. 61,1 % ohne bDMARDs.

## Diskussion

Die hier vorgelegten Daten in einer Population von Patienten mit entzündlich rheumatischen Erkrankungen zeigen eindeutig, dass im Hinblick auf das Vorhandensein von protektiven Antikörpern gegen Masern ein nicht zu vernachlässigender Teil der Patienten nicht ausreichend gegen Masern geschützt ist. Unsere Daten zeigen allerdings auch, dass in der Mehrzahl der Fälle weder die Erhebung der Impfanamnese noch die Erhebung der Infektionsanamnese die Identifikation der ungeschützten Risikopopulation ermöglicht. Diese ließ sich v. a. durch die Bestimmung der Masernantikörper im Blut identifizieren. Als praktische Konsequenz sollten unsere Ergebnisse eine Verbesserung in der Erkennung von Lücken im Masernimpfstatus bei Patienten mit entzündlich rheumatischen Erkrankungen erzielen.

Bei der häufigsten entzündlich rheumatischen Erkrankung, der rheumatoiden Arthritis, sind häufig ältere Menschen betroffen. Vor vielen älteren Masernerkrankten in der Bevölkerung wird auch wegen der nicht selten schweren Verläufe gewarnt [34, 35]. Daher ist es wichtig, dass es auch in älteren Altersgruppen keine Lücken der Immunität gibt. Die Immunität von Erwachsenen ist nicht nur für die Unterbrechung der Übertragung von Bedeutung, sondern auch dafür, schwere Erkrankungen zu verhindern. Das ist für ältere Patienten mit rheumatischen Erkrankungen von potenziell erheblicher Bedeutung. Allerdings zeigen unsere Daten, dass bei älteren Patienten kein Nachlassen der Masernimmunität gemessen an der Höhe des Masernantikörpertiters vorliegt. Wie sich die Höhe der Masernantikörpertiter bei der nach 1970 geborenen Bevölkerung auswirken wird, sollte Gegenstand der weiteren Forschung bleiben. Hier zeigen unsere Daten, dass die Höhe des Masernantikörpertiters bei geimpften Personen niedriger ist als bei geimpften Personen, was zum größten Teil an der fehlenden zweiten Auffrischimpfung liegen dürfte.

Wenn es in einer Bevölkerung genug immune Individuen gibt, können Masernausbrüche verhindert werden [36]. Für die Erreichung einer sog. „Herdenimmunität“ müssen am besten 95 % der Menschen immun gegen Masern sein [6, 8, 37]. Basierend auf einer Reproduktionszahl von 11 bei Masern – das ist die Anzahl der Sekundärfälle, die von einer typischen Infektion in einer völlig anfälligen Bevölkerung ausgehen bzw. verursacht werden –, muss sichergestellt sein, dass mindestens 85 % der 1‑ bis 4‑Jährigen, 90 % der 5‑ bis 9‑Jährigen und 95 % der 10-Jährigen und Älteren nachweisbar immun gegen Masern sind [1–3, 8]. Die Immunitätsniveaus, die für die Eliminierung von Masern aktuell für notwendig erachtet werden, sind höher als die bisherigen Leitlinien. Obwohl ein solch hohes Niveau schwer zu erreichen sein kann, bietet z. B. der Schulbeginn die Gelegenheit, systematisch ausreichende Immunität zu gewährleisten. Darüber hinaus muss v. a. für einen weitreichenden Schutz der älteren Bevölkerung gesorgt werden, und hier v. a. für die immunkompromittierten Menschen.

Der MMR-Impfstoff ist eine Mischung von in ihrer Virulenz abgeschwächten Viren, die per Injektion zur Immunisierung gegen Masern, Mumps und Röteln eingesetzt wird. Geimpft werden im deutschsprachigen Raum generell Kinder im Alter von etwa 1 Jahr, mit einer Zweitimpfung im zweiten Lebensjahr. Bei Einhaltung dieses Impfschemas ergibt sich ein Schutz von über 99 % gegen diese Infektionskrankheiten. Die Immunität gilt als sehr lange andauernd, sehr wahrscheinlich ein Leben lang – ohne dass eine Auffrischung benötigt wird. So konnte bei Personen, die gegen Masern, Mumps und Röteln geimpft wurden, gezeigt werden, dass diese zumeist auch nach 20 Jahren noch ausreichend hohe Antikörpertiter besitzen [20, 38, 39]. In unseren Daten konnten wir keine Abnahme der Antikörperspiegel über die Zeit beobachten. Die ist jedoch eher durch das parallele Auftreten von Antikörperspiegeln aufgrund einer Wildtypmaserninfektion und Antikörperspiegeln durch eine Masernimpfung erklärbar ist, wobei die Wildtypinfektion höhere Spiegel induziert.

Laut RKI kann bei positivem Nachweis von IgG-Antikörpern gegen MV grundsätzlich von Immunität, d. h. von Schutz gegen Masern, ausgegangen werden [8, 11, 12]. Für die Bestimmung der Masernantikörper, und zwar unabhängig, ob nach Impfung oder nach durchgemachter Erkrankung, lassen sich mindestens 2 relevante Testverfahren unterscheiden: der Plaque Reduction Neutralisation-Test (PRNT) und ein Enzyme-Linked Immunosorbent Assay (ELISA). Der PRNT gilt international als Goldstandard, misst er doch unmittelbar die für die humorale Immunität letztendlich entscheidenden neutralisierenden Antikörper, er ist aber technisch und zeitlich sehr aufwendig und arbeitet darüber hinaus mit lebenden Masernviren, was eine Standardisierung erschwert [40]. Der ELISA ist im Vergleich zum PRNT schneller, kostengünstiger, automatisierbar und leichter zu standardisieren, weshalb er sich in den letzten Jahren in den meisten europäischen Labors als Standardmethode der Antikörperbestimmung nicht nur bei Masern durchgesetzt hat. Der Test ist aber nicht sehr sensitiv, d. h. er entdeckt eine bestehende Immunität weniger zuverlässig als der PRNT, weil die damit gemessenen IgG-Antikörper nicht unmittelbar mit der tatsächlich vorhandenen Immunität korrelieren, sie sind nur ein Surrogat der Immunität. Bei fehlendem Nachweis von Antikörpern im ELISA nach einer Masernimpfung ist mit dem PRNT oft noch Immunität nachweisbar. Quantitative Vergleiche der beiden Tests zeigten, dass mehr als die Hälfte der im ELISA negativen Personen im PRNT positiv reagierten, d. h. doch eine Immunität gegen Masern aufwiesen [41, 42]. Unsere Ergebnisse, die mit der ELISA-Technik gewonnen worden sind, könnten also potenziell mit einem aufwendigeren Test noch weiter verbessert werden. Ob die unterschiedlichen Antikörpertiter von Geimpften und natürlich Infizierten eine klinische Rolle spielen, ist noch Gegenstand der wissenschaftlichen Diskussion.

Das Thema Impfen bei Immundefizienz ist im letzten Jahr vom RKI ausführlich behandelt worden und wird an anderer Stelle ausführlich thematisiert [24].

Zusammengefasst ist der Impfschutz gegen Masern bei Patienten mit rheumatischen Erkrankungen noch unzureichend. Obwohl es bisher keine Berichte über eine größere Zahl von Masernfällen bei diesen Patienten gibt, ist ein sicherer Impfschutz auch unter dem Aspekt einer ausreichenden Herdenimmunität zu gewährleisten. Die neue Gesetzeslage ermöglicht es und verpflichtet die Rheumatologen, dafür zu sorgen, dass möglichst alle Patienten mit rheumatischen Erkrankungen gegen Masern geschützt sind. Das zu erreichen ist sicher nicht trivial, v. a. angesichts der immer noch zu zahlreichen Impfgegner.
